# Activity seascapes highlight central place foraging strategies in marine predators that never stop swimming

**DOI:** 10.1186/s40462-018-0127-3

**Published:** 2018-06-21

**Authors:** Yannis P. Papastamatiou, Yuuki Y. Watanabe, Urška Demšar, Vianey Leos-Barajas, Darcy Bradley, Roland Langrock, Kevin Weng, Christopher G. Lowe, Alan M. Friedlander, Jennifer E. Caselle

**Affiliations:** 1 Department of Biological Sciences, Florida International University, North Miami, Florida USA; 2 National Institute of Polar Research, Tachikawa, Tokyo Japan; 3 Department of Polar Science, SOKENDAI (The Graduate University for Advanced Studies), Tachikawa, Tokyo Japan; 4 School of Geography and Sustainable Development, University of St Andrews, St Andrews, Scotland UK; 5 Department of Statistics, Iowa State University, Ames, Iowa USA; 6 Bren School of Environmental Science and Management, University of California Santa Barbara, Santa Barbara, California USA; 7 Department of Business Administration and Economics, Bielefeld University, Bielefeld, Germany; 8 Department of Fisheries Science, Virginia Institute of Marine Science, College of William & Mary, Gloucester Point, Virginia USA; 9 Department of Biological Sciences, California State University Long Beach, Long Beach, California USA; 10 Department of Biology, University of Hawaii at Manoa, Honolulu, Hawaii USA; 11 Pristine Seas, National Geographic Society, Washington DC, USA; 12 Marine Science Institute, University California Santa Barbara, Santa Barbara, California USA

**Keywords:** Sharks, Acceleration, Hidden Markov models, Coral reefs, Foraging, Telemetry

## Abstract

**Background:**

Central place foragers (CPF) rest within a central place, and theory predicts that distance of patches from this central place sets the outer limits of the foraging arena. Many marine ectothermic predators behave like CPF animals, but never stop swimming, suggesting that predators will incur ‘travelling’ costs while resting. Currently, it is unknown how these CPF predators behave or how modulation of behavior contributes to daily energy budgets. We combine acoustic telemetry, multi-sensor loggers, and hidden Markov models (HMMs) to generate ‘activity seascapes’, which combine space use with patterns of activity, for reef sharks (blacktip reef and grey reef sharks) at an unfished Pacific atoll.

**Results:**

Sharks of both species occupied a central place during the day within deeper, cooler water where they were less active, and became more active over a larger area at night in shallower water. However, video cameras on two grey reef sharks revealed foraging attempts/success occurring throughout the day, and that multiple sharks were refuging in common areas. A simple bioenergetics model for grey reef sharks predicted that diel changes in energy expenditure are primarily driven by changes in swim speed and not body temperature.

**Conclusions:**

We provide a new method for simultaneously visualizing diel space use and behavior in marine predators, which does not require the simultaneous measure of both from each animal. We show that blacktip and grey reef sharks behave as CPFs, with diel changes in activity, horizontal and vertical space use. However, aspects of their foraging behavior may differ from other predictions of traditional CPF models. In particular, for species that never stop swimming, patch foraging times may be unrelated to patch travel distance.

**Electronic supplementary material:**

The online version of this article (10.1186/s40462-018-0127-3) contains supplementary material, which is available to authorized users.

## Background

Central place foraging (CPF) is a ubiquitous behavior seen across animal groups ranging from insects, to birds, and humans [1]. Unlike random movements within a home range, CPF behavior consists of periodic and predictable movements to and from a central place, often with multiple individuals sharing the central place [1]. CPF animals tend to rest at the central place, with their energy costs increasing as they travel greater distances from this location [1, 2]. As such, the costs associated with travel distance to the patch should define the limits of the animals foraging range from the central place [2, 3]. CPF behavior can lead to heterogeneity in habitat or prey distribution as the animal’s foraging rates will likely vary with distance from the central place [4, 5]. As patch distance to the central place increases, travel costs also increase, and the animal should spend more time foraging at the patch [1, 4].

A key assumption of CPF theory is that an animal rests at the central place, and foraging costs increase with travel distance to a feeding patch. Yet, there are many species of marine predators that exhibit CPF-like behavior, but never stop swimming, and never truly rest. For these animals, energy costs may be independent of travel distance to the patch, and simply a function of swim speeds. Such predators include large coral reef-associated fishes (sharks and teleosts), which swim within a relatively small core area during the day, and move over an expanded area at night, with periodicity of movements related to diel or tidal cycles [6–9]. Marine animals also move in a three-dimensional (3D) environment and CPF behavior can include a vertical component as well as a horizontal one, with individuals performing diel vertical migrations (DVM) relative to the central place [10–12]. While few studies have measured actual activity and swim speeds, some tropical reef sharks display patterns of activity that also vary with diel and tidal cycles [13–17]. However, why predators that do not use a shelter or ever stop swimming require a central place, is unclear.

CPF animals that never stop swimming are almost exclusively ectotherms, so metabolic rates are sensitive to changes in ambient temperature. At any particular time, routine metabolic rates should be a function of body temperature, movement speed, and other aspects of the movement process (e.g. turning costs more than straight line swimming [18]). While the animal may not stop swimming, they can establish the central place in cooler waters where metabolic rates are reduced. If the animal simultaneously maintains low activity in the central place while cooler, then energy costs may be essentially similar to ‘resting’ [10]. In addition to changes in body temperature, routine metabolic rates can be modulated via changes in swim speed. Hence, the energetics of CPF in these animals must consider body temperature and movement rates.

Accelerometers have become popular for measuring both the activity and energy requirements of free-ranging marine animals [14, 19–21]. Accelerometer data can be combined with information about the geographic location of the animal to generate a spatial representation of the animal’s energy costs [19–21]. However, it is difficult to separate areas of high-energy expenditure (e.g. traversing through an energetically expensive habitat) from areas of high animal activity related to specific behaviors (e.g. foraging) within the landscape, especially for animals whose behaviors cannot be easily defined from sensor measurements (e.g. continuously swimming fish). For our general case of CPF foragers, we are interested in how the predator’s foraging activity varies spatially in relation to the central place. This challenge is further complicated in fishes because space use and activity are often measured at different temporal scales, due to limitations of tagging technology. Movements can be recorded via telemetry over multiple years, while fine-scale behaviors from accelerometers are recorded over time-frames of days to weeks. The diel behavior of CPF animals is likely to be predictable, suggesting that measurements made over shorter time periods are representative of long-term behavior [14]. We took advantage of this predictability to develop a new spatial representation of activity we term an ‘activity seascape’, for marine predators which show CPF behavior. The activity seascape combines long-term patterns of space use derived from acoustic telemetry data with the diel probability of being more or less active, which is based on statistical time-series models inferred from short deployment accelerometers [22]. The seascapes allow the locations/times where high activity may be strongest/weakest to be identified without requiring space use and activity of individuals to be measured simultaneously.

We use multi-sensor loggers and statistical time-series models to build activity seascapes for two species of reef shark (blacktip reef sharks (*Carcharhinus melanopterus*), and grey reef sharks (*C. amblyrhynchos*)), whose movements appear similar to a CPF as they use smaller areas during the day than at night [7–9, 12, 14, 23]. We also used these activity seascapes to better understand the dynamics of CPF in animals that never stop swimming. Specifically, we predict that sharks use a central place during the day while in deeper and cooler water where they decrease activity and reduce energy costs. We predict they move over a larger area at night into shallower water, where they become more active. We then build a simple bioenergetics model for grey reef sharks to predict the relative contribution of body temperature and swim speed to daily energy budget. We further predict that grey reef sharks will maintain lower body temperatures within their central place as a tactic to reduce ‘travel costs’ within their refuge.

## Methods

### Study site

Research was conducted at Palmyra Atoll (5°54’ N; 162°05’ W), located in the central Pacific Ocean. Palmyra has been a U.S. federal wildlife refuge since 2001, with only a research station housing a small number of scientists and staff. The atoll consists of two lagoons, surrounded by sandflats and backreefs, which transition to forereef habitats (see Additional file 1: Appendix S1). Due to its protected status, large shark populations are found at the atoll [24]. Further details of the site can be found in [9, 14].

### Patterns of activity

#### Data-loggers

The first component of our study relates to how shark activity varies throughout the diel and tidal cycles, and how it varies by depth and water temperature. In order to quantify behavior, we tagged 5 blacktip and 5 grey reef sharks with external data-loggers (ORI400-D3GT loggers, 12-mm diameter, 45-mm length, and 9 g; Little Leonardo Co., Tokyo, Japan) in July 2013. Sensors measured 3D acceleration (sampled at 20 Hz), swimming depth, water temperature (1 Hz), and some animals were also fit with a speed sensor (1 Hz, three grey reef sharks) and video camera (one blacktip, two grey reef sharks, Table 1). We used DVL400 video cameras (recording duration 5–11 h) that recorded at 640 × 480 pixels at 30 frames/second. The video cameras were programmed to turn on the day after the animal was released, to avoid the period of stress associated with tagging. Cameras turned on at 07:00–08:00 and recorded continuously until the battery ran out (5 h for the blacktip, 11 h each for the grey reef sharks, with the difference due to camera battery size). Loggers and cameras were embedded in copolymer foam floats attached to the dorsal fin via tie wraps, and a time-release mechanism caused the package to detach 3–5 days after deployment. Tags floated to the surface where an embedded VHF transmitter enabled us to locate and retrieve them.

**Table 1:** Details of sharks tagged with data-loggers or acoustic transmitters. BLT (blacktip reef shark), GR (grey reef shark), TL (Total Length)

Tag Type	BLT	TL range (cm)	Sex (M:F)	GR	TL range (cm)	Sex (M:F)
1) Data-logger	4	113–127	0:4	4	149–159	0:4
Speed	2	113, 127		3	154–159	
Video	1	127		2	154–158	
2) Acoustic transmitters	20	105–133	0:20	43	107–168	13:30
Depth/temperature	9	105–126		13	107–168	4:9
Acceleration/depth	6	105–133		0		

#### Analysis of shark activity

We filtered the static contribution of gravity from raw acceleration data, and then calculated overall dynamic body acceleration (ODBA, [25]). ODBA was used as a measure of activity as it incorporates tail beat frequency as well as activity along other body axes. However, being time series data, ODBA values are highly correlated such that inferences based on statistical models that do not take this key feature into account will usually be invalid. Furthermore, the autocorrelation structure itself will provide interesting behavioral information as the probability of the animal being active is likely to be a function of how active it was previously. Understanding the biological importance of changes in ODBA in animals that swim continuously is difficult as there is no true ‘resting’ period. We were interested in periods of increased activity, particularly ‘bursts’ which could be indicative of foraging behavior.

Hidden Markov models (HMMs) are stochastic time series models where observed data (e.g. travel speed, depth, ODBA) are assumed to be driven by an underlying hidden process. We assume that the hidden process can be in either of *N* = 2 states, roughly corresponding to behavioral states, which we label ‘relatively high activity’ and ‘relatively low activity’, respectively. The observations can be considered noisy measurements of the behavioral state, which cannot be explicitly observed [22, 26]. Traditionally, HMMs have inferred behavioral states from the movement process itself (e.g. rate of movement, turning angles), but more recently have been used with behavioral data and ODBA specifically [22]. We developed a 2-state HMM, where sharks were in either a low state of activity (*state 1*) or high state of activity (*State 2*), based on ODBA data from accelerometers. We could then compute probabilities of sharks changing or remaining within behavioral states based on time of day, tidal state, or swimming depth and water temperature. ODBA values were averaged over 1 s intervals before applying the HMMs, based on observations of behaviors from video footage (see below). We removed the first 4 h of data from each animal so that we did not infer behavior when the animals may still have been highly stressed. All HMMs were built in the statistical environment R using customized code. All HMM details can be found in Additional file 2: Appendix S2.

The HMMs provide a data-driven, objective approach to analyzing acceleration data, but we still cannot identify which specific behavior (e.g. feeding, predator avoidance) sharks perform while within the various states. We had the unique opportunity to correlate the results of the HMM with the simultaneously collected 22 h of video obtained from two grey reef sharks. Video data allowed us to see what the sharks were doing (and in which habitats) when inferred by the HMM to be in certain states, providing validation for our interpretation of the model. We first observed all 22 h of video noting times of foraging, increased activity (both of the individual shark and conspecifics) as well as the presence of other sharks. For those specific time periods, we could then compare observations with the behavioral state predicted by the HMM.

We also analyzed swim speed data in grey reef sharks as a direct measure of energy expenditure. To explore diel changes in swim speed, we constructed generalized additive models (GAMs) for each individual grey reef shark (*N* = 3) using hourly mean swim speed data. Speed will still likely suffer from serial correlation, which we accounted for by including an AR(1) (first-order auto-regressive) process with time as the position variable. The correlation at lag = 1 was included in the model to specify the correlation structure. The GAM was constructed with a Gaussian error distribution and time of day was modeled with a cyclic smooth spline. Model fit was assessed by examining residual diagnostic plots, and Akaike’s information criterion [27] (AIC) was used to assess model performance against a null model (intercept only), with improved model fit indicated by a minimum ΔAIC value > 3 [28]. GAM analyses were conducted in R using the *mgcv* package.

### Patterns of space use

#### Acoustic telemetry

We quantified reef shark space use patterns using acoustic telemetry methods. Between 2010 and 2014, sharks were caught on hook and line and surgically implanted with an acoustic transmitter (V16, 69 kHz, Vemco ltd, Nova Scotia). A small incision was made on the ventral surface, the transmitter was inserted into the body cavity and a single suture was used to close the incision. The sharks were measured, sexed, externally dart tagged and released. These individuals were not the same sharks as those tagged with data-loggers above, although there was temporal overlap between periods of data collection (i.e. individuals were being tracked during the same time when sharks were carrying loggers). We acoustically tagged a total of 20 blacktip reef sharks, and 43 grey reef sharks. Of those, 9 blacktip reef sharks and 13 grey reef sharks were tagged with V16 PT transmitters that also measured pressure (depth) and body temperature, and 6 blacktip reef sharks were tagged with V13 AP transmitters that measured 3D acceleration in addition to depth. Transmitters were detected by an array of up to 70 subsurface omni-directional acoustic receivers (VR2W) deployed throughout the atoll [14]. Every time a shark swam within range of the receiver, the date/time of detection, along with swimming depth and body temperature (for those individuals with PT tags) were recorded. We downloaded receivers annually. Receiver detection range can vary by habitat and range tests of a subsample of receivers showed an approximate range of 250 m on backreefs and close to 500 m on forereef habitats. Note that blacktip reef shark acceleration, depth, and body temperatures were analyzed in [14].

We calculated shark spatial utilization distributions (UD) using a Brownian bridge movement model (BBMM), where consecutive acoustic detections between receivers by moving sharks are linked by conditional random walks [29, 30]. The BBMM also incorporates measurement error (we set this to 300 m as the average receiver detection range) to provide a more realistic representation of the possible space used by the animal [30]. CPF behavior will consist of periodic excursions from the central place, and we were interested in the distances sharks moved throughout the diel period from this core location. We calculated the central place for each individual separately. For blacktip reef sharks, we defined the central place as the 50% UD predicted by the BBMM. However, grey reef sharks were detected on far fewer receivers, making it difficult to calculate 2-dimensional BBMMs. As such, for each individual we defined the core area as the receiver where 80% of detections were made, with a 1000 m buffer (assuming a detection range of 500 m on either side of forereef receivers). For both species of shark, we then quantified the distance of movement from this core area throughout the diel cycle. That is, for each individual we created a time series of distances to its own central place and then in the last step we averaged these distances into a time series of average displacement for each shark species. Analyses were performed in R using the *adehabitat LT* and *adehabitat HR* packages.

To explore diel patterns of vertical habitat use by grey reef sharks, we constructed generalized additive mixed models (GAMMs) using hourly mean depth and body temperature data from the acoustic transmitters. Specifically, we estimated the effect of time of day on swimming depth and temperature, with individual shark modeled as a random effect. All other model components were the same as described above for swim speed. Diel changes in depth and temperature for blacktips were reported in [14].

#### Patterns of space use and activity seascapes

The acceleration data and HMMs provided probabilities of sharks being in a relatively high active state throughout the diel cycle. We then combined these with three-dimensional UDs calculated over months to years of movement from the telemetry data, to generate an overall visual representation of the ‘activity seascape’ of individual animals within their environment. We used the telemetry data to produce space-time cubes (STC) where the bottom two dimensions represent the geographic space over which the movement occurs and the third dimension is time [29]. In a 2D kernel density UD, the surface representing this density is divided into square grid cells or pixels, the value of which represents the probability of movement in that particular location. Analogously, in a 3D UD, the space-time volume is divided into so-called voxels, i.e. three-dimensional grid cubes, with each voxel assigned a probability of movement in that particular location in space and time. Our three-dimensional BBMM generalizations allow us to use time as part of the BBMM calculation, and show time visually on the third axis of the space-time density volume [29]. We built 3D UDs for each shark by aggregating telemetry data by day. The space-time UDs were then combined with the diel activity probabilities from HMMs in order to visually emphasize areas in the space-time UDs when sharks were the most active. We did this by multiplying each voxel in the space-time UD volume with the probability at that particular moment in time of the shark being in an active state, as determined by the HMMs. We removed any individuals from analysis that had < 100 detections. The activity seascape algorithm is in the process of being published as R package. In the mean time, a preliminary version of the R code will be placed to https://github.com/udemsar upon publication.

### Bioenergetic model

To predict the relative contribution of swimming speed and body temperature on daily energy budgets, we built a simple bioenergetics model for grey reef sharks. We calculated shark mass using W = 0.0045 L^3.21^ where L is total length in cm and W is weight in kg (http://fishbase.org). Routine metabolic rates (M) were then estimated using the equation for ectothermic sharks, Log_10_M = 0.79*Log_10_W + 2.31 [31]. Metabolic rates were corrected for changes in body temperature assuming a Q_10_ of 1.65 and 3.0, to include the range of Q_10_ values seen within tropical sharks [32, 33]. To predict the effect of changing swim speed, we used the model developed for requiem sharks in [34]. Briefly, we assume that the average swimming speed for each animal represents its optimal travel speed that minimizes its cost of transport. The percentage increase or decrease in swim speeds from this average leads to an equivalent change in metabolic rate (e.g. 1% increase in speed = 1% increase in active metabolic rate [31]). We calculated hourly changes in routine metabolic rate for a 38.3 kg (average grey reef shark body mass at Palmyra) individual using the observed diel changes in body temperature, and then assuming the animal maintained constant body temperature throughout the diel cycle (average 28.0 °C). Diel changes in swim speed were inferred from the GAM results from the swim speed sensor data, described above. Model results were compared with a paired 2 sample t-test. Our goal was not to estimate daily energy costs, but rather to determine if observed diel changes in body temperature were biologically relevant (at least regarding energy costs) and the relative contribution of shark swim speed to the daily energy budget.

## Results

### Patterns of activity

**Fig. 1:**
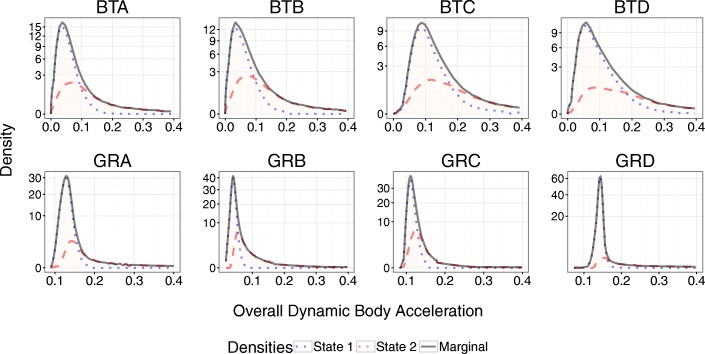
State dependent conditional densities of Overall Dynamic Body Acceleration (ODBA) values for blacktip reef sharks (BTA-D) and grey reef sharks (GRA-D). Sharks were fitted with accelerometers for periods of 3–5 days. *State 1* is a low activity state, while *state 2* is a high activity state. The y-axis is the density of the estimated state-dependent probability density functions, weighted by the proportion of observations that correspond to each state. The marginal density is the sum of the weighted-state dependent densities

**Fig. 2:**
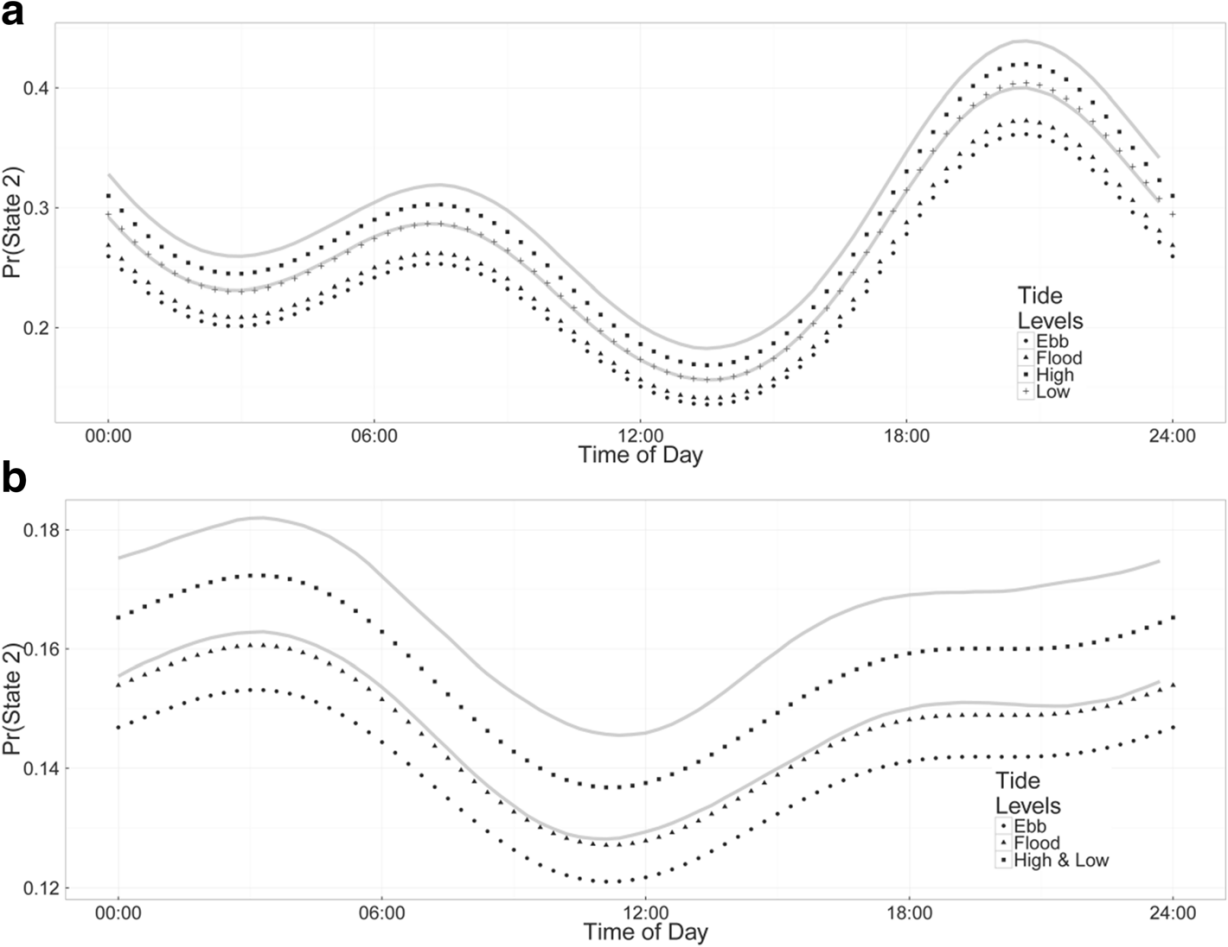
Diel and tidal changes in the probability of blacktip reef (**a**) and grey reef sharks (**b**) being in a relatively high activity state (*state 2*). Probabilities were calculated by applying a hidden Markov model to acceleration data. The solid grey lines represent 95% confidence intervals for one tidal state (high tide). For clarity, other tidal confidence intervals have not been included

We recovered behavioral information from data-loggers deployed on four blacktip reef sharks (120 ± 6 cm Total Length (TL), all female, 16 days) and four grey reef sharks (155 ± 5 cm TL, all females, 15 days, Table 1). Hidden Markov models were constructed considering two behavioral states; *state 1* (‘relatively low activity’) was associated with fairly constant relatively low levels of ODBA, while *state 2* (‘relatively high activity’) involved overall higher activity levels and in particular included any bursts in ODBA (Fig. 1). The HMMs identified clear diel patterns in activity for both species, although the patterns and magnitude of the differences varied (Fig. 2). Blacktip reef sharks showed higher overall probabilities of being in *state 2* at night with a clear peak between 20:00–21:00 and lowest activity in the early afternoon (12:00–15:00). Probability of high activity during the day was as low as 15% and increased to an early evening probability of 40%, depending on tide (Fig. 2a). Grey reef sharks showed the highest probability of activity (*state 2*) from approximately 21:00–06:00, with a peak at 03:00 (Fig. 2b). Lowest levels of activity occurred between 11:00–12:00. However, the probability of grey reef sharks being in a high activity state was only 17% at night (during high or low tide), with a minimum 12% probability in the late morning (during the ebb tide) (Fig. 2b). Overall, sharks were in the low activity state (*state* 1) for the majority of their time (blacktip reef sharks 77.7% range 71.8–83.6%, grey reef sharks 86.1% range 76.3–96.7%). Blacktip reef sharks remained in *state 2* for 19–26 s (means), while grey reef sharks were in *state 2* for 10–29 s bursts.

**Fig. 3:**
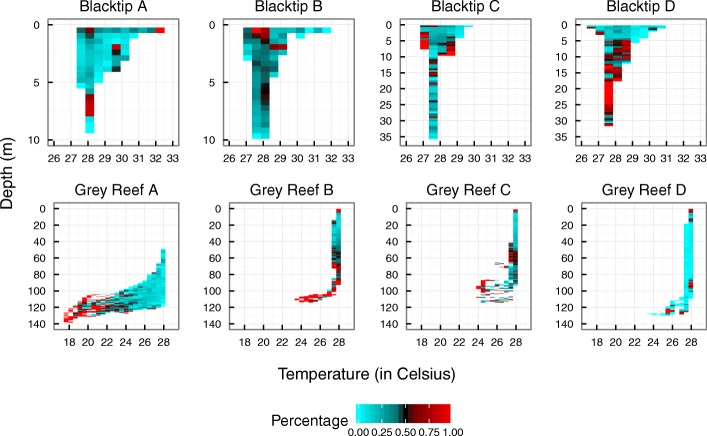
Vertical behavior of female reef sharks at Palmyra atoll. Swimming depth and water temperature has been colour coded based on the percentage of time sharks were in a high activity state (*state* 2). Behavioral state was predicted by hidden Markov model from acceleration data. Red designates a high proportion of time spent in *state 2* at that depth/temperature

HMMs also revealed significant variation in behaviour with depth (Fig. 3). Blacktips used relatively shallow depths with the majority of time spent < 10 m, although two individuals made brief single dives to 30 m (Fig. 3, Additional file 3: Appendix S3). During deeper dives, sharks were in an active state (*state 2*) but overall, individuals were most active at depths of 4–10 m, or at the surface if temperatures were 28 °C. All sharks were in a low activity state (*state 1*) when they were at the surface in waters > 29.5 °C (Fig. 3). The depth distribution for grey reef sharks was variable, with animals primarily using depths in the 30–80 m range, but occasionally diving as deep as 120 m, although one individual rarely went shallower than 80 m (see Additional file 3: Appendix S3). Sharks showed a bimodal pattern in the probability of being in *state 2*, spending a high percentage of time active when in shallow water but also when performing brief deep dives below the thermocline, which was located at approximately 100 m (Fig. 3).

**Fig. 4:**
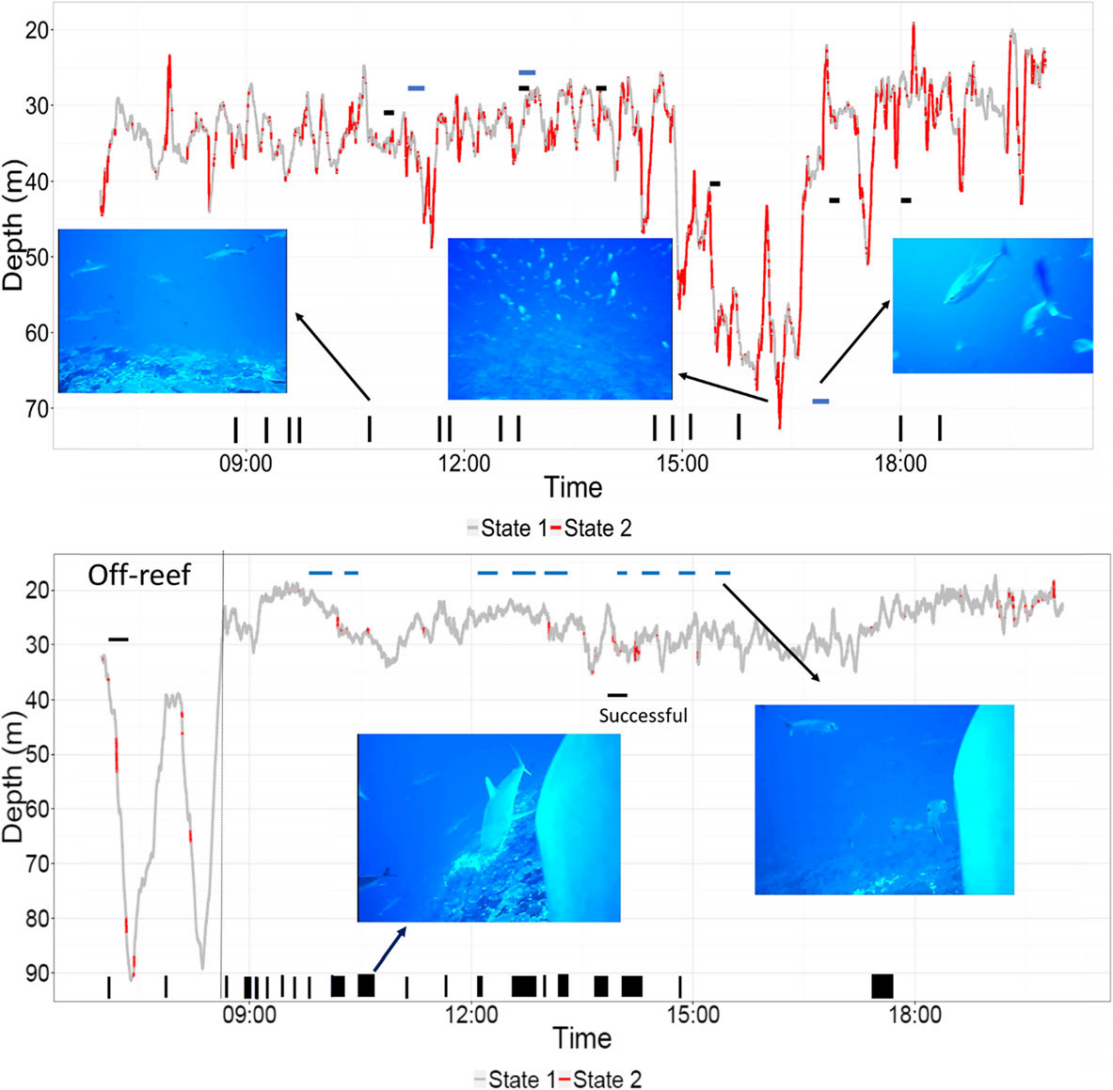
A day in the life of grey reef shark B (upper) and D (lower) as determined by multi-sensor data-loggers. Depth has been color coded to indicate the behavioral state; low activity (*state 1*) or high activity (*state 2*), using acceleration data and HMMs. Video cameras were used to observe behaviors. Horizontal black dashes indicate foraging attempts or success. Vertical black bars indicate when the sharks associated with con-specifics. In upper panel, horizontal blue lines indicate when yellowfin tuna were seen foraging on reef. Horizontal blue lines on lower panel highlight when the shark was associating with schooling barracuda. Note the high abundance of fishes on the mesophotic reef (70 m) in shark B

We obtained 22 h of video footage from two grey reef sharks, which revealed that both sharks used forereef habitats during the day although shark D was offshore (pelagic) during the early morning hours (Fig. 4). Foraging attempts were defined by the shark accelerating towards the reef followed by lateral snapping of the head and reef fishes in proximity displaying anti-predator maneuvers. Two foraging attempts, one of which was successful (sinking scales were seen after the attempt, Additional file 4: Appendix S4), were observed by shark D, while seven attempts were observed for shark B. The HMM predicted individuals to be in *state 2* for all foraging attempts. Other grey reef sharks (and sometimes whitetip reef sharks, *Triaenodon obesus*) were frequently seen in the footage, with > 10 individuals in frame at multiple points in time (Fig. 4). With one exception, if sharks were in *state 2* while other sharks were seen, then the other individuals were also highly active (i.e. multiple individuals were being active) and in some cases appeared to be foraging (Additional file 5: Appendix S5). Shark D was frequently seen associating with schools of great barracuda (*Sphyraena barracuda*). On three occasions, yellowfin tuna (*Thunnus albacares*) were observed foraging on the reef near shark B (Fig. 4). Shark B used mesophotic reefs (> 50 m) with very high reef fish abundance, including schooling planktivorous species.

Grey reef sharks (154–159 cm TL) swam at average speeds of 0.51–0.63 m/s with maximum speeds of 3.6 m/s. Although the average speeds differed between the three individuals, they all showed the same diel pattern with increased speeds at 02:00–03:00 and lowest speeds from 12:00–18:00 (Additional file 6: Appendix S6). Average swim speeds increased by 5–10% during the night (GAM AIC values = − 13,561.7.27 (A), − 10,896.4 (C), − 27,936.8 (D) compared to null models AIC values = − 8423.6 (A), − 7730.9 (C), − 16,824.0 (D), Additional file 5: Appendix S5).

### Patterns of space use and activity seascapes

**Fig. 5:**
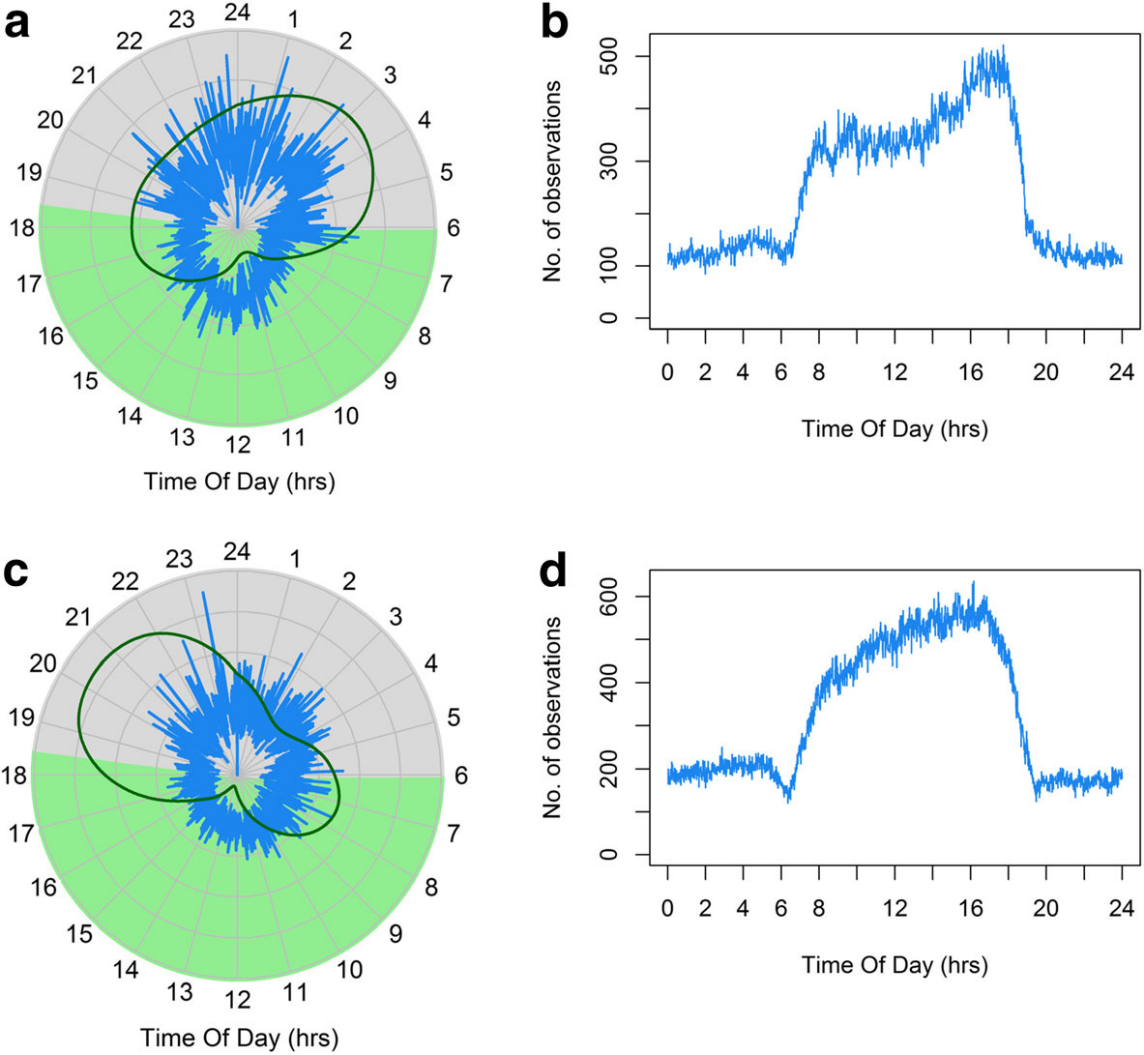
Diel changes in average displacement from the central place by blacktip reef (**a**, *n* = 20) and grey reef sharks (**c**, n = 20). Grey and light green backgrounds show night and day respectively. Probabilities of sharks being in *state 2* (high activity) are shown as dark green lines (redrawn from Fig. 2). There were diel differences in number of detections (**b**, **d**) so daytime estimates of distance are based on larger sample sizes. Blacktip reef shark nocturnal displacements averaged 1 km, while grey reef sharks averaged 3 km from the central place

**Fig. 6:**
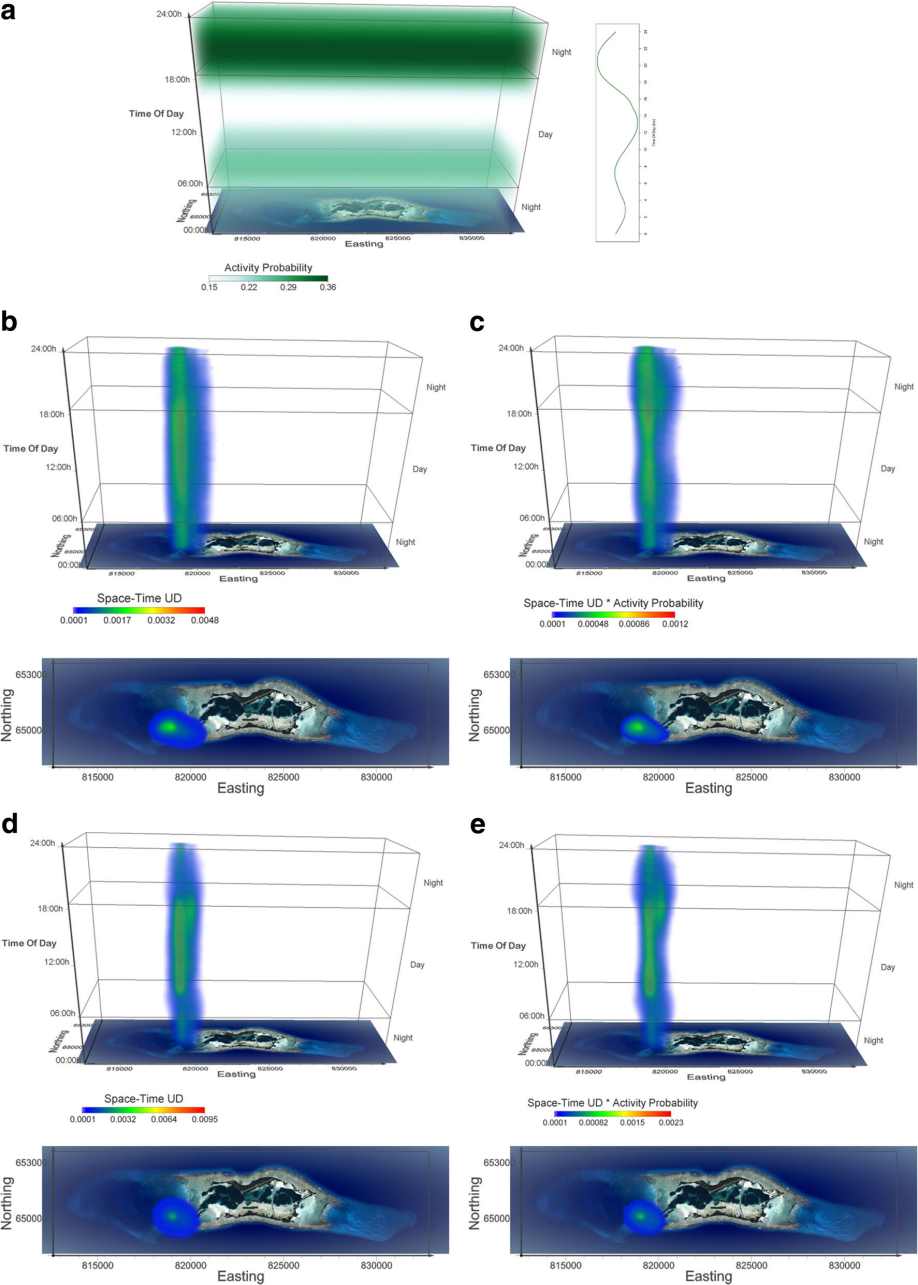
Activity seascapes for blacktip reef sharks at Palmyra atoll. **a** The volume of probability of diel activity determined from accelerometers (see Fig. 2) shown in a space-time cube, where each voxel gets the value of respective probability based on the time in the voxel. Darker/lighter green voxels in the probability volume correspond to higher/lower probability values in the chart. **b** Space-time density volume for blacktip reef sharks (*n* = 17). The bottom panel shows the projection of the volume onto the two geographic dimensions, i.e. the map of Palmyra atoll. Note that this is a view from above the 3D UD volume and not a 2D UD surface. **c** Activity seascape for blacktip reef sharks (n = 17), where voxel values are obtained as a voxel-by-voxel product of volumes in **a**) and **b**). The bottom panel shows the projection of the volume onto the map of Palmyra atoll. **d** Space-time density for an individual blacktip reef shark (duration = 1108 days) and **e**) activity seascape for the same blacktip reef shark. Note the similarity of the individual patterns with the respective patterns of the whole group in panels **b**) and **c**). Voxel size in all volumetric representations is 100 m × 100 m × 10 min, creating a Space-Time volume of 202 × 56 × 144 voxels (20.2 km × 5.6 km × 24 h)

**Fig. 7:**
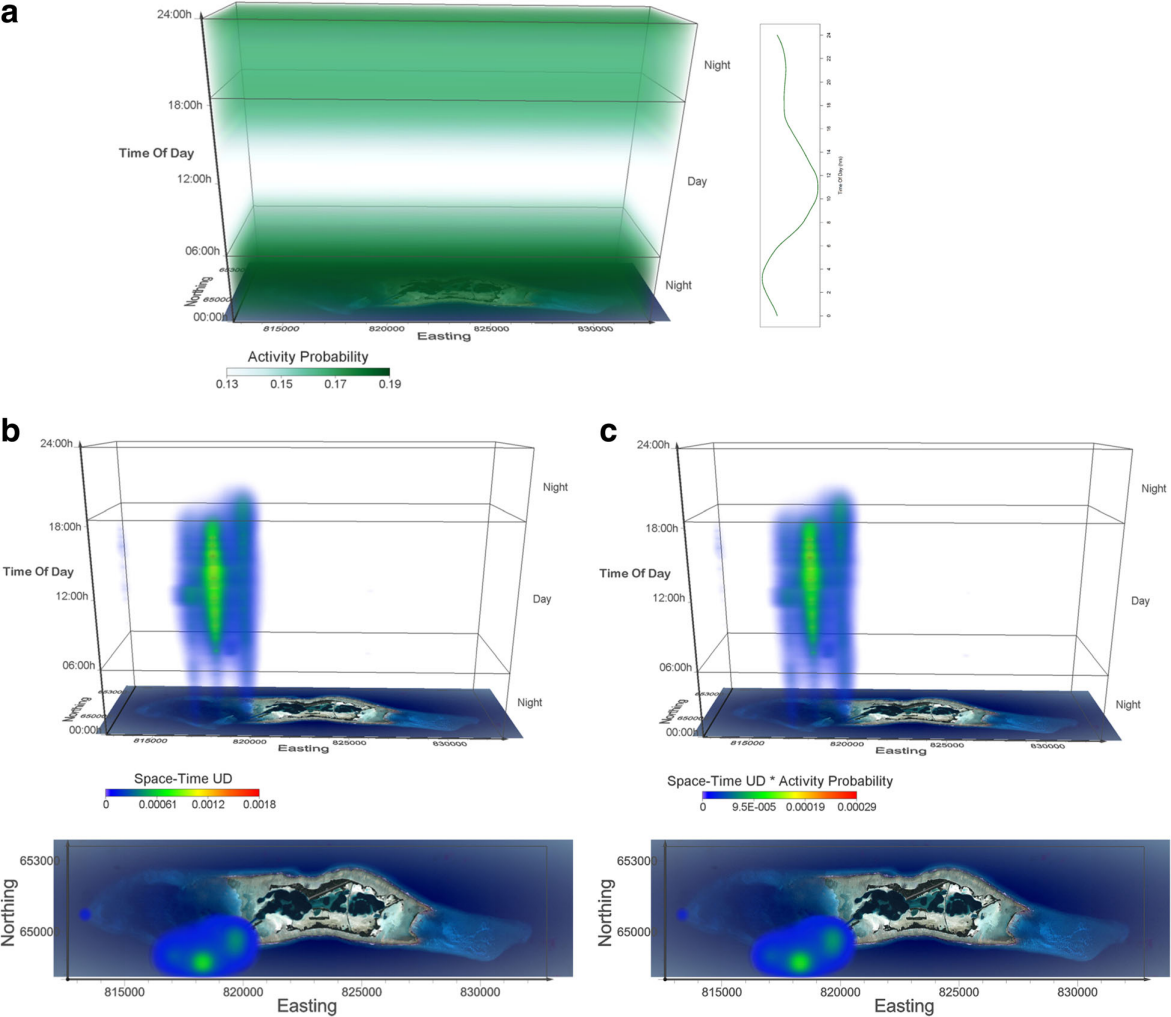
Activity seascapes for grey reef sharks at Palmyra atoll. **a** The volume of probability of diel activity determined from accelerometers shown in a space-time cube, where each voxel gets the value of respective probability based on the time in the voxel. Darker/lighter green voxels in the probability volume correspond to higher/lower probability values in the chart. **b** Space-time density volume for grey reef sharks (n = 17). The bottom panel shows the projection of the volume onto the two geographic dimensions, i.e. the map of Palmyra atoll. Note that this is a view from above the 3D UD volume and not a 2D UD surface. **c** Activity seascape for grey reef sharks (n = 17). The bottom panel shows the projection of the volume onto the map of Palmyra atoll. Voxel size in all volumetric representations is 100 m × 100 m × 10 min, creating a Space-Time volume of 202 × 56 × 144 voxels (20.2 km × 5.6 km × 24 h)

We acoustically tagged 20 female blacktip reef sharks (119 ± 7 cm TL) on the backreefs at Palmyra. Sharks were detected for an average of 392 ± 341 days (range 22–1108), although individuals detected for short durations were tagged with transmitters with shorter battery lives (e.g. acceleration transmitters). The majority of individuals (70%) showed evidence of limited diel movement patterns, using a core area during the day and moving over an expanded activity space at night (Figs. 5a and 6). However, the remaining 30% of individuals appeared to use the same areas day and night. Blacktip reef sharks had activity spaces of 1.9 ± 2.7 km^2^ and had diel displacements from the core area of 0.73 ± 0.63 km. We acoustically tagged and detected 43 grey reef sharks (30 F: 13 M, 143 ± 17 cm TL) for an average of 1012 ± 429 days (range 5–1545 d) on the Palmyra forereefs. 92.5% of individuals showed diel movement patterns, being detected on the forereefs during the day with far fewer detections at night, although we also captured the night time habitat use of some individuals (Figs. 5c, d and 7). Grey reef sharks had activity spaces of 4.4 ± 1.3 km^2^ and showed daily displacement of 2.9 ± 1.3 km. We note that activity space and displacement estimates are only representative of reef shark movements within the receiver array and are therefore likely underestimates of both. For both species, sharks showed excursions from the central place that increased in distance between sunset and sunrise, although there were far fewer detections at night (Fig. 5). The nighttime excursions were far more pronounced in grey reef sharks than blacktip reef sharks, which only showed extended movements during the early evening when they were also predicted to be most active (Fig. 5). Acoustic noise is also louder at night (e.g. snapping shrimp), which will likely reduce the number of acoustic detections as well (K. Weng unpublished data).

Shark vertical habitat use appeared to vary relative to the diel cycle and the area of core use. Our previous analysis of blacktip reef shark vertical movements and body temperature (from acoustic telemetry data), revealed very moderate diel changes, with these sharks using water 2–3 m deeper during the day [14]. Grey reef sharks displayed more dramatic diel shifts in swimming depths, using deeper depths during the day (average 53 m) than at night (average 23 m, GAMM, AIC value = 193,034.1, null model AIC value = 206,293.0, Additional file 7: Appendix S7). Body temperatures in grey reef sharks also varied with the diel period, with lower body temperatures during the day (average 27.8 °C) when the sharks were deeper, than at night (average 28.1 °C, GAMM, AIC value = 11,059.5, null model AIC value =15,518.0, Additional file 7: Appendix S7), although there was more variability between individuals compared to diving patterns.

Due to our limited sample size of data-logger equipped sharks, and to ensure that shark movement data (from telemetry) at least overlapped temporally with the period of data-logger deployment, we only generated activity seascapes for blacktip reef sharks tagged in the backreef and grey reef sharks tagged along the SW portion of the atoll (locations where data-logger equipped sharks were caught and released). As such, activity seascape data consists of 17 blacktip reef sharks tagged on the backreefs and 17 grey reef sharks tagged within and just outside the channel (Additional file 1: Appendix S1). Activity seascapes for both species showed a high concentration of space use/activity in the daytime central place, with activity becoming spatially more diffuse at night (Figs. 6 and 7). For both species, multiple individuals used the same daytime core area, suggestive of refuging behavior (Figs. 6 and 7). As changes in diel activity were more pronounced in blacktip reef sharks, activity seascapes showed greater variation from simple space use UDs. The importance of daytime activity was greatly reduced within the central place, while the early evening use of the main channel was highlighted as a location where activity of blacktip reef sharks may be high (Fig. 6).

### Bioenergetic model

**Fig. 8:**
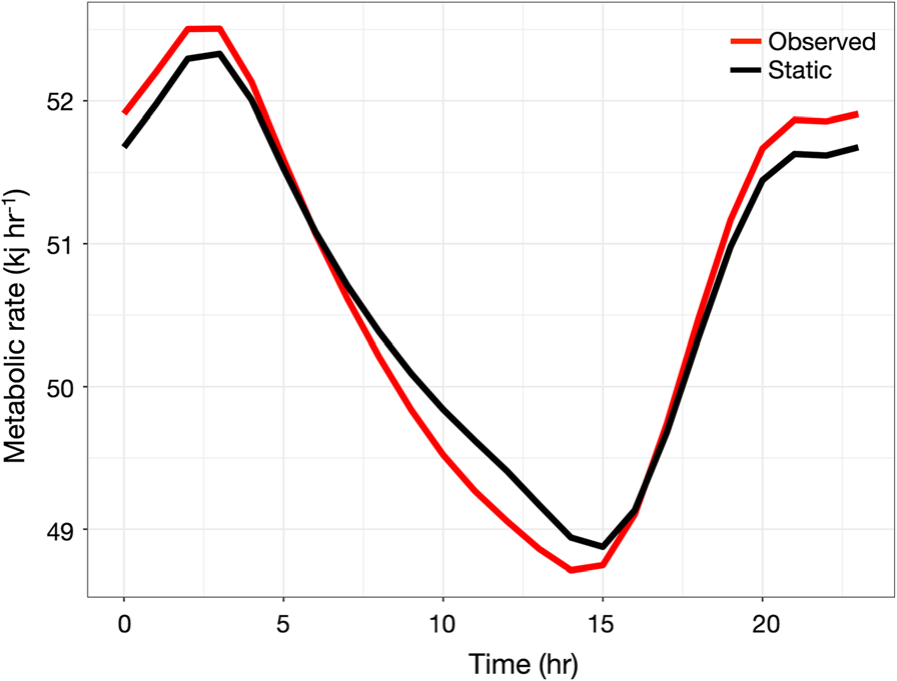
Predicted diel changes in routine metabolic rates for a 38.3 kg grey reef shark, based on body temperature and swim speed. ‘Observed’ uses actual diel changes in body temperature, while ‘static’ assumes a constant body temperature of 28 °C throughout the diel cycle

There were clear diel changes in estimated routine metabolic rates due to the nocturnal increase in swim speed (Fig. 8). Overall, there was an approximately 7% difference in routine metabolic rate between the early morning (02:00–3:00) and the early afternoon (12:00–15:00). However, there was very little difference in predicted energy expenditure between an animal with a 0.5 °C diel change in body temperature, and one maintaining a constant body temperature throughout the diel cycle, regardless of the Q_10_ (paired t-test for means, Q_10_ = 1.6 *t* = 0.09, *p* = 0.93, Q_10_ = 3.0 *t* = − 0.00067, *p* = 0.99).

## Discussion

As predicted, both species of shark used a smaller central place in deeper water during the day when they were less active, before expanding movements into shallow water at night with a concurrent increase in activity. Blacktip reef sharks showed more dramatic diel changes in activity (but much more subtle changes in swimming depth) and were most active during the early evening, while grey reef sharks tended to be most active in the middle of the night. The early evening peak in activity seen in blacktip reef sharks was previously suggested to be a function of increased foraging success (due to falling or low light levels) and predator/prey thermal physiology [14]. Aspects of CPF behavior clearly vary between the species, as blacktip reef sharks use the central place day and night; they just expand the total area they use at night. Grey reef sharks were rarely detected at their central place at night so were not reusing the same area throughout the diel cycle. Furthermore, there is strong spatial separation between the species at Palmyra, with blacktip reef shark activity spaces located in the backreef, lagoons, and shallow forereef, while grey reefs are situated on the deeper sections of the forereefs [35]. Activity cycles are based on small samples sizes, although there is evidence that diel activity rhythms in tropical reef sharks fit a more general pattern [23, 36]. For example, an acoustic camera placed in the main channel at Palmyra documented a peak in number of sharks (assumed to be blacktip reef sharks based on nighttime fishing) seen at the exact time and place we predict maximal blacktip reef shark activity (early evening in the channel [37]).

Changes in activity were concurrent with diel vertical migrations to and from the central place. Blacktip reef sharks perform small shifts in swimming depth, but tides played a greater role in swimming depth, with individuals moving close to the surface during daytime low tide periods, where they are directly warmed by the sun [14, 38, 39]. Grey reef sharks maintained cooler body temperatures during the day by occasionally diving below the thermocline and showed a greater diel shift in depth. However, their diel variation in body temperature was small and dives below the thermocline during the day were short and tended to occur while they were in a high activity state. Due to thermal inertia effects, grey reef sharks would have warmer muscle temperatures than mesophotic teleost prey residing below the thermocline, as long as dive durations are short. The increased muscle performance from being warmer could provide sharks with a hunting advantage during these periodic deeper excursions. Although behavior below the thermocline was consistent between individual grey reef sharks, there was also clearly some variability in the depths selected by individuals, with one shark staying considerably deeper than the others. The reasons for this variability are unknown, but could reduce intra-specific competition which is also thought to drive horizontal separation between grey reef shark groups at Palmyra [35].

Activity seascapes and video footage suggest that *some* foraging may occur in the central place of grey reef sharks at Palmyra. If foraging has some significance to CPF behavior in grey reef sharks, then we would expect the location of the central place to be in an area of high diurnal prey biomass. We recently conducted a mark-recapture study to determine spatially dependent densities for grey reef sharks at Palmyra [24]. All fishing was performed during the day so technically these distribution patterns represent daytime central place locations. We also used diver surveys to estimate spatial patterns of biomass for reef fishes of lengths 10–60 cm, as a proxy of potential prey distribution ([40], NOAA Coral Reef Ecosystem Program, Additional file 8: Appendix S8). Qualitatively, there was broad overlap between shark density hotspots and prey biomass, with highest levels of both metrics at the eastern and western tips and lower values along the southern and northern portions of the atoll (Additional file 8: Appendix S8). While abiotic conditions may explain these patterns (e.g. areas of high current flow), we at least provide evidence that grey reef shark central place locations are in areas where prey is most abundant. Similarly, seabirds establish central place locations dependent on local productivity and prey availability, although of course seabird prey are not located directly at the central place [41].

Although our results match our general predictions, our bioenergetics model for grey reef sharks suggests that body temperatures are having an insignificant effect on routine metabolic rates, at least at the diel scale. Instead, diel changes in routine metabolic rates are driven by changes in swim speed. Similarly, activity made a much larger contribution to the short-term field metabolic rates of sea snakes than changes in temperature [21]. These results suggest that CPF behavior of grey reef sharks may involve some different mechanisms than those seen in other animals, at least for the Palmyra population (i.e. individuals at other locations may display more dramatic diel changes in body temperatures). Firstly, we cannot assume that foraging does not take place within the central place, which can have implications for patterns of intra-specific competition in refuging animals. Secondly, energy costs will not be a function of travel distance to the central place, but instead related to movement speeds and likely tortuosity [18]. Unlike seabirds and other colonial CPF, access to foraging patches by sharks should not be a function of distance to the patch as they still have to swim within the central place; sharks simply modulate speed [3]. This would also call into question a key prediction of traditional CPF models; in reef sharks foraging time within a patch should be independent of patch to central place distance, in species that never stop swimming [4]. Of course, this assumes the only currency of value to the sharks is energy, when time itself may also be important, especially if coral reefs are heterogeneous with regards habitat quality. In this case, the time taken to swim to distant patches may come at lost opportunities of foraging at more reliable patches close to the central place.

Why would sharks, which do not use a nest/shelter or stop swimming, need a central place? CPF would require some degree of memory although the animal may only have to use basic path integration and memorization of simple landmarks to return to a central place, which should optimize foraging rates [42, 43]. Therefore, CPF behavior may optimize foraging success via improved navigation to foraging areas, while minimizing memory requirements (which come with costs) when prey communities are relatively abundant [42, 43]. CPF behavior may also foster social associations between reef sharks, as ‘meeting’ would require a common daytime habitat. We found that multiple individuals used the same core daytime areas, and video footage showed large numbers of conspecifics sharing the same areas and at times even foraging simultaneously. It is becoming increasingly apparent that some species of shark, including grey and blacktip reef sharks, are capable of forming social associations with conspecifics, and ‘refuging’ during the day may be a mechanism to allow such associations to form [6, 15, 44, 45].

Ideally, one would record spatial position and high resolution activity from each individual simultaneously, and over long time periods, improving our ability to identify ‘activity hotspots’ [19]. Doing so with fishes will require technological advancements to existing satellite or acoustic telemetry techniques or the development of completely new technologies [20, 21]. While cyclical patterns of activity are likely reasonably consistent in CPF animals that show high residency, this may change throughout the year and requires testing. For example, a semi-captive study with lemon sharks suggested that the extent of diel activity differences could be a function of sex and social position [15]. New logging technologies may also enable feeding events and meal size to be directly measured in free-ranging sharks, rather than inferred from acceleration signals [46]. Analytically, state space models and HMMs can now incorporate multiple variables simultaneously from individual animals (e.g. diving depth, acceleration, stomach temperature, turn angles) to infer and validate behavioral states and produce population-level activity budgets [47, 48]. Future studies could also then address individual variability in behavior rather than homogenizing it within population scale analyses.

Understanding the small-scale dynamics of predation requires approaching behavior from a spatial and temporal context in concert. When considering predator effects on prey, we must consider patterns of predator habitat selection, but also predator *behavior* within those habitats. A recent study suggested that even trophic cascades in marine systems may be confined to spatial ‘hotspots’ used by predators (e.g. [39]). Our activity seascape approach simultaneously displays the space used and diel activity of marine predators and could further identify locations of ecological importance, taking into account space use *and* behaviour. These advancements could significantly improve our understanding of how marine predators may influence lower trophic levels and further explain the evolution of CPF behavior in marine animals.

## Conclusions

We develop a new method for simultaneously displaying diel space use and activity, in marine animals where space use and behavior are often measured over different time scales. We use this method to show that reef sharks behave similar to central place foragers, occupying deeper water during the day where they are less active, and being more active in shallower water at night. However, unlike other CPF animals, grey reef sharks at least, showed some evidence of foraging in the central place. Furthermore, diel changes in energy expenditure appear to be regulated by changes in swim speed and not body temperature. The implications are that traditional predictions from CPF theory regarding patch foraging times, may not apply for CPF animals that never stop swimming.

## Additional files

Additional file 1: **Appendix S1.** Map of Palmyra atoll and locations of acoustic receivers. (DOCX 645 kb)

Additional file 2: **Appendix S2.** Details of the hidden Markov model built to analyze shark accelerometer data. (DOCX 15 kb)

Additional file 3: **Appendix S3.** Swimming depth distribution of individual blacktip reef and grey reef sharks fitted with data-loggers. (DOCX 23 kb)

Additional file 4: Video footage from a grey reef shark showing a foraging event on the reef. (WMV 10244 kb)

Additional file 5: **Appendix S5.** Table showing percentage time grey reef sharks were in an active state for specific behaviours, determined from animal-borne video cameras. (DOCX 12 kb)

Additional file 6: **Appendix S6.** Diel changes in swim speed for three grey reef sharks fitted with swim speed sensors. Both average changes in speed and a cyclic spline from a generalized additive model are shown. (DOCX 325 kb)

Additional file 7: **Appendix S7.** Diel changes in swimming depth and body temperature for grey reef sharks at Palmyra atoll (*n* = 13) as determined by acoustic telemetry. The y-axis are the standardized residuals from a generalized additive mixed model. Dashed lines indicate 95% confidence interval around the smooth term. (DOCX 230 kb)

Additional file 8: **Appendix S8.** Spatially dependent densities for grey reef sharks, determined from a mark-recapture study (Bradley et al. 2017, lower panel) and ‘potential prey’ abundance determined by diver surveys (upper panel, NOAA, CREP 2015). (DOCX 409 kb)
