# Supplementary material for: Activity seascapes highlight central place foraging strategies in marine predators that never stop swimming
Source: Mov Ecol. 2018 Jun 21;6:9. doi: 10.1186/s40462-018-0127-3 (PMC6011523; doi:10.1186/s40462-018-0127-3)
Supplement: Supplementary file 1 — Appendix S1. Map of Palmyra atoll and locations of acoustic receivers. (DOCX 645 kb) [file 40462_2018_127_MOESM1_ESM.docx]

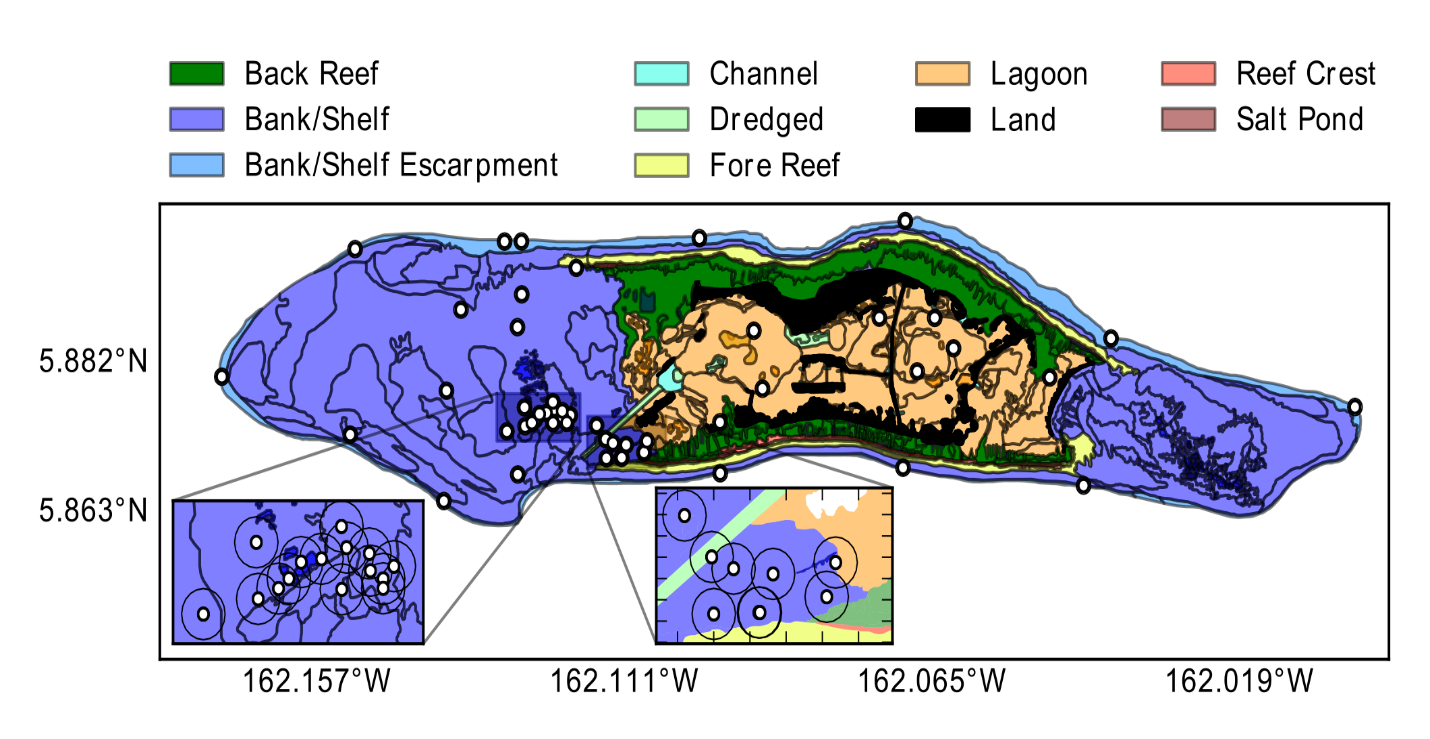


Additional file 1: Appendix S1. Palmyra atoll with habitat designations and the locations of acoustic listening stations (VR2Ws).
