# Supplementary material for: Activity seascapes highlight central place foraging strategies in marine predators that never stop swimming
Source: Mov Ecol. 2018 Jun 21;6:9. doi: 10.1186/s40462-018-0127-3 (PMC6011523; doi:10.1186/s40462-018-0127-3)
Supplement: Supplementary file 2 — Appendix S2. Details of the hidden Markov model built to analyze shark accelerometer data. (DOCX 15 kb) [file 40462_2018_127_MOESM2_ESM.docx]

Appendix S2: Details of hidden Markov model

The observable state-dependent process is denoted by $\left\{ Y_{t} \right\}_{t=1}^{T}$ (e.g. ODBA) while an unobservable state process is denoted $\left\{ S_{t} \right\}_{t=1}^{T}$ (Langrock et al. 2012, Leos-Barajas et al. 2017). The observations are assumed to be conditionally independent given the states and we assume a first-order Markov process for the states (i.e. the state at time $t$ only depends on the state at time $t$-1). The evolution of the behavioural states over time is governed by a nonhomogeneous transition probability matrix, t.p.m., $(t)=\gamma_{ij}(t)$ , where $\gamma_{ij}(t)=$ Pr($S_{t+1}=j |S_{t}=i$) for $i,j=1,\ldots,N$ and $N$is the number of states (where we assume $N$=2). The vector of initial state probabilities, $\boldsymbol{\delta}$, where $\delta_{i}=\Pr\left( S_{1}=i \right),i=1,\ldots,N,$ denotes the probabilities of the shark being first observed in either of the $N$ states, which we estimated. The state-dependent densities, $f_{k}$ for $k=1,\ldots,N$, were estimated nonparametrically using a P-splines approach, with $f_{k}$ expressed as a finite linear combination of basis functions

$$f_{k}\left( y_{t} \right)=\sum_{m=1}^{M} a_{k,m} \phi_{k,m}\left( y_{t} \right)$$

and a penalty term added to the likelihood to control for smoothness. The basis functions $\phi_{k,m}$ are known and fixed probability density functions, thus only requiring that the mixing proportions $a_{k,m}$ be estimated, subject to ${0\leq a}_{k,m}\leq1$ and $\sum_{m}^{M} a_{k,m}=1$. The nonparametrically estimated state-dependent densities provide a flexible framework that can accommodate multiple forms, thereby eliminating the need to select a family of distributions, e.g. gamma, normal or Weibull distributions. We decided on this form for $f_{k}$ as a preliminary analysis showed that standard parametric HMMs did not fit the data well. Further, we allowed for shark-specific state-dependent densities to account for individual differences in ODBA values and assumed common t.p.m. parameters within species. The entries of the t.p.m. were given as a function of diel and tidal components in the following manner in order to infer their effects on the state-switching behaviour:

$$\gamma_{ij}\left( t \right)= \beta_{0i}+\beta_{1i}\cos\left( \frac{2\pi t}{86400} \right)+ \beta_{2i}\sin\left( \frac{2\pi t}{86400} \right)+ \beta_{3i}\cos\left( \frac{2\pi t}{43200} \right)+ \beta_{4i}\sin\left( \frac{2\pi t}{43200} \right)+ \beta_{5i}x_{t,high}+ \beta_{6i}x_{t,flood}+ \beta_{7i}x_{t,ebb}$$

for $i,j=1,2, i\neq j$, with $x_{t,high}=1$if high tide was observed at time $t$ and equal to zero otherwise, similarly for $x_{t,flood}$ and $x_{t,ebb}$, and $\beta_{0i}$ corresponded to ebb tide. The value of 86,400 reflects the number of seconds in a day, with corresponding cosine and sine terms denoting a diel cycle, and 43,200 corresponding to a 12-hour cycle. We calculated the HMM likelihood using the forward algorithm, which allows for parameter estimation via a numerical maximum likelihood approach. Given the maximum likelihood estimates for the parameters of the HMM, we used the Viterbi algorithm to decode the optimal state sequence underlying each time series, thus connecting each observation to one of the $2$ states (Zucchini, MacDonald, & Langrock 2016). ODBA values were averaged over 1 sec intervals before applying the HMMs based on observations of behaviours from video footage (see below). To determine how activity varied by swimming depth and water temperature, we related the decoded state sequences to a grid of depth and temperature values. For each grid cell (~ 0.5 m x 0.5° C), we calculated the percentage of decoded states corresponding to state 1 or state 2.

References:

Langrock, R., King, R., Matthiopoulos, J., Thomas, L., & Morales, J.M. (2012) Flexible and practical modelling of animal telemetry data: hidden Markov models and extensions. *Ecology*, **93**, 2336-2342.

Leos-Barajas, V., Photopoulou, T., Langrock, R., Patterson, T.A., Watanabe, Y.Y, Murgatroyd, M., & Papastamatiou, Y.P. (2017) Analysis of animal accelerometer data using hidden Markov models. *Methods in Ecology and Evolution*, **8**, 161-173.

Zucchini, W., MacDonald, I.L., & Langrock, R. (2016) *Hidden Markov models for time series: an introduction using R, 2^nd^ edition.* Chapman & Hall/CRC, Boca Raton, FL, USA
