# Supplementary material for: Activity seascapes highlight central place foraging strategies in marine predators that never stop swimming
Source: Mov Ecol. 2018 Jun 21;6:9. doi: 10.1186/s40462-018-0127-3 (PMC6011523; doi:10.1186/s40462-018-0127-3)
Supplement: Supplementary file 3 — Appendix S3. Swimming depth distribution of individual blacktip reef and grey reef sharks fitted with data-loggers. (DOCX 23 kb) [file 40462_2018_127_MOESM3_ESM.docx]

Depth use by four blacktip reef sharks at Palmyra Atoll (a-d corresponds with BTA-BTD). Sharks were fitted with depth data-loggers for 4 days each.

Depth use by four grey reef sharks (a-b corresponds to GRA-GRD) at Palmyra Atoll. Sharks were fitted with depth data-loggers for 3-6 days each.
