# Supplementary material for: Activity seascapes highlight central place foraging strategies in marine predators that never stop swimming
Source: Mov Ecol. 2018 Jun 21;6:9. doi: 10.1186/s40462-018-0127-3 (PMC6011523; doi:10.1186/s40462-018-0127-3)
Supplement: Supplementary file 5 — Appendix S5. Table showing percentage time grey reef sharks were in an active state for specific behaviours, determined from animal-borne video cameras. (DOCX 12 kb) [file 40462_2018_127_MOESM5_ESM.docx]

|  | Shark B (%) | Shark D |
| --- | --- | --- |
| Increased activity | 21 | 4 |
| Other grey reef sharks | 37.5 | 2 |
| Trevally | 17 | - |
| Barracuda | - | 6 |

Percentage of time individual sharks were classified in *state 2* (high activity) by the hidden Markov model, relative to what was seen by the shark-borne cameras. These included some increase in activity without any evidence of foraging, other grey reef sharks in frame, and either barracuda or bigeye trevally.
