# Supplementary material for: Activity seascapes highlight central place foraging strategies in marine predators that never stop swimming
Source: Mov Ecol. 2018 Jun 21;6:9. doi: 10.1186/s40462-018-0127-3 (PMC6011523; doi:10.1186/s40462-018-0127-3)
Supplement: Supplementary file 6 — Appendix S6. Diel changes in swim speed for three grey reef sharks fitted with swim speed sensors. Both average changes in speed and a cyclic spline from a generalized additive model are shown. (DOCX 325 kb) [file 40462_2018_127_MOESM6_ESM.docx]

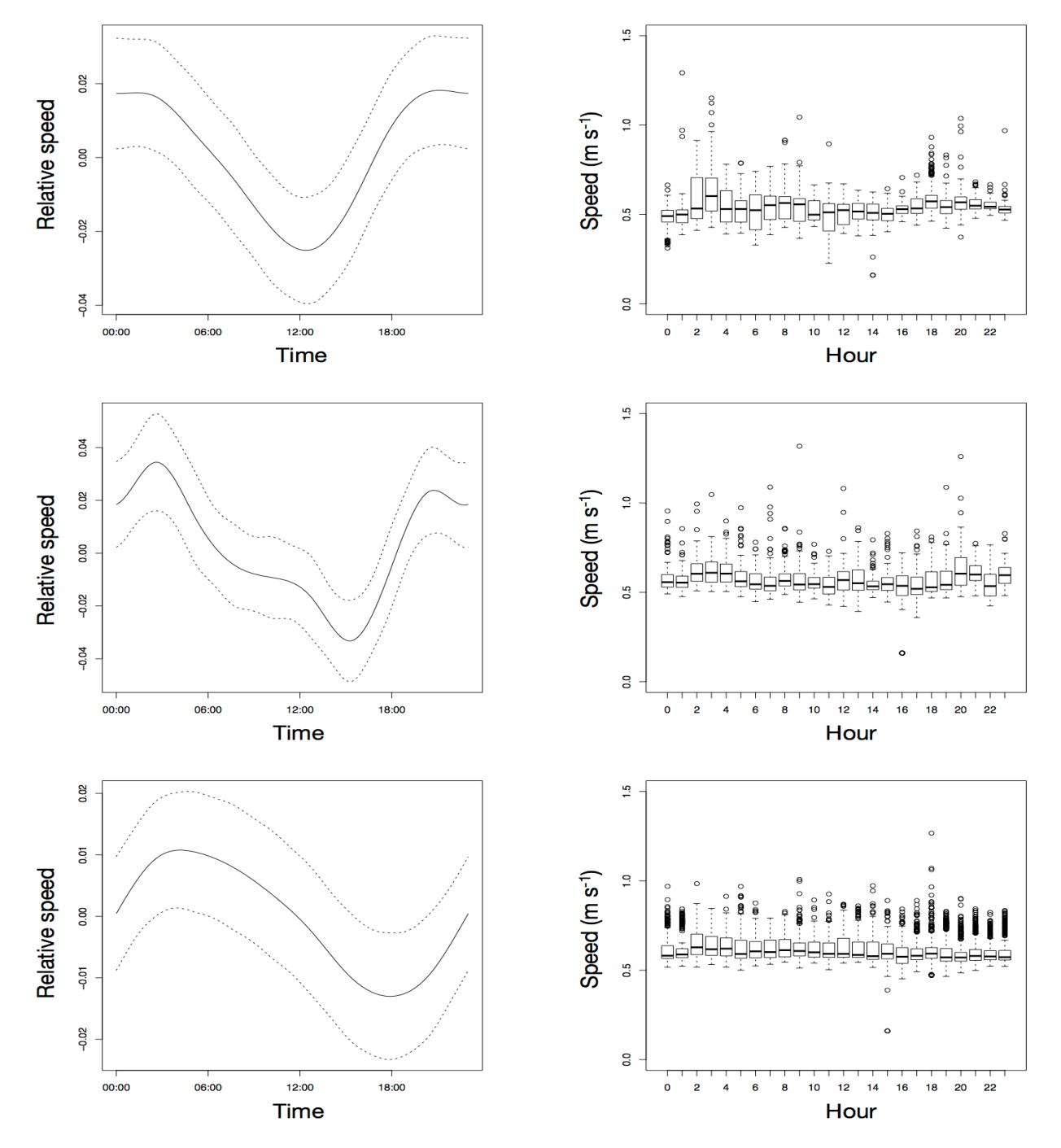


Diel changes in swim speed for three grey reef sharks fitted with swim speed sensors. Both average changes in speed and a cyclic spline of residuals from a generalized additive model are shown.

| Shark | TL (cm) | Swim speed (cm/s) | AIC (time) | AIC (intercept) | F | p | R^2^ |
| --- | --- | --- | --- | --- | --- | --- | --- |
| 1 | 158 | 0.54 | -13561.7.3 | -8423.6 | 1.92 | <0.001 | 0.045 |
| 2 | 154 | 0.57 | -10896.4 | -7730.9 | 4.65 | <0.002 | 0.066 |
| 3 | 159 | 0.62 | -27936.8 | -16824.0 | 1.03 | 0.009 | 0.022 |

Average swim speeds and GAM results for three grey reef sharks fitted with swim speed sensors.
