# Supplementary material for: Activity seascapes highlight central place foraging strategies in marine predators that never stop swimming
Source: Mov Ecol. 2018 Jun 21;6:9. doi: 10.1186/s40462-018-0127-3 (PMC6011523; doi:10.1186/s40462-018-0127-3)
Supplement: Supplementary file 7 — Appendix S7. Diel changes in swimming depth and body temperature for grey reef sharks at Palmyra atoll (n = 13) as determined by acoustic telemetry. The y-axis are the standardized residuals from a generalized additive mixed model. Dashed lines indicate 95% confidence interval around the smooth term. (DOCX 230 kb) [file 40462_2018_127_MOESM7_ESM.docx]

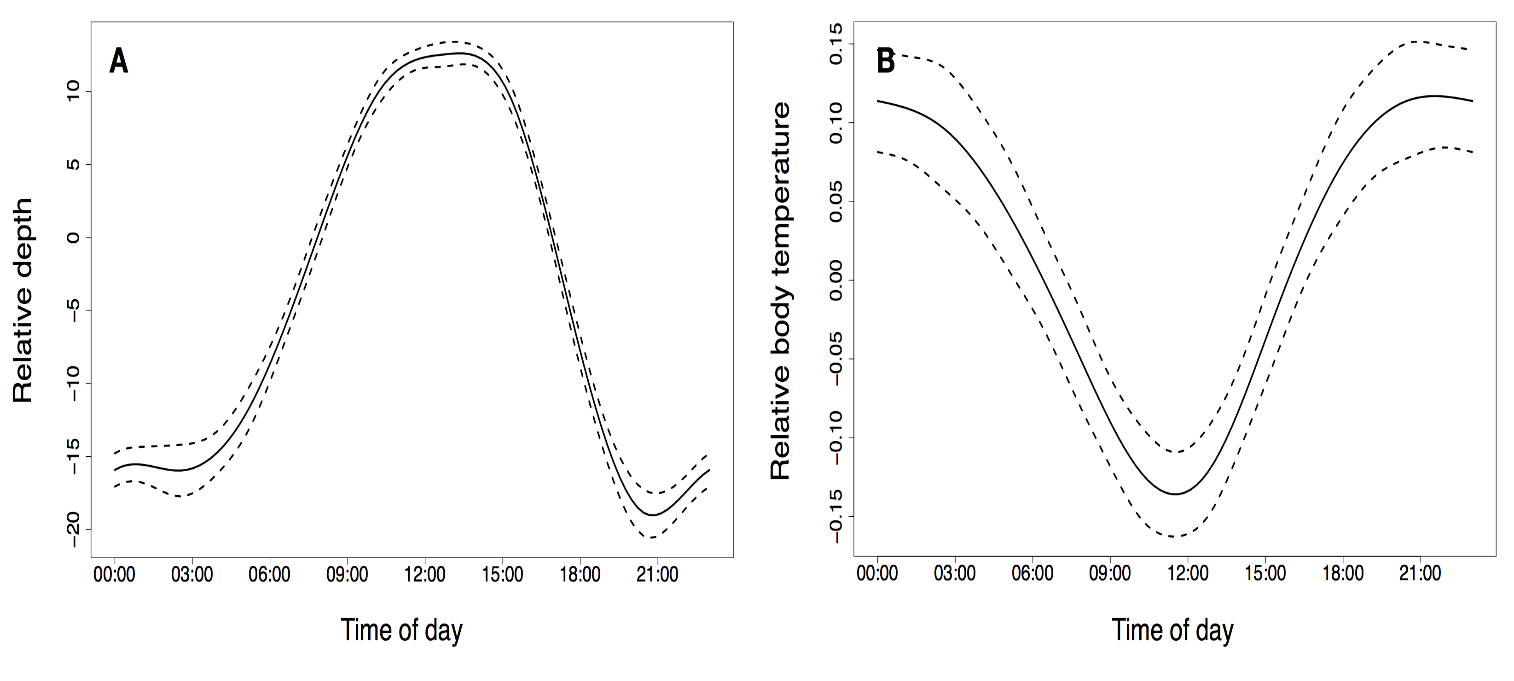


Appendix S7: Diel changes in swimming depth and body temperature for grey reef sharks at Palmyra atoll (n=13) as determined by acoustic telemetry
