# Supplementary material for: Activity seascapes highlight central place foraging strategies in marine predators that never stop swimming
Source: Mov Ecol. 2018 Jun 21;6:9. doi: 10.1186/s40462-018-0127-3 (PMC6011523; doi:10.1186/s40462-018-0127-3)
Supplement: Supplementary file 8 — Appendix S8. Spatially dependent densities for grey reef sharks, determined from a mark-recapture study (Bradley et al. 2017, lower panel) and ‘potential prey’ abundance determined by diver surveys (upper panel, NOAA, CREP 2015). (DOCX 409 kb) [file 40462_2018_127_MOESM8_ESM.docx]

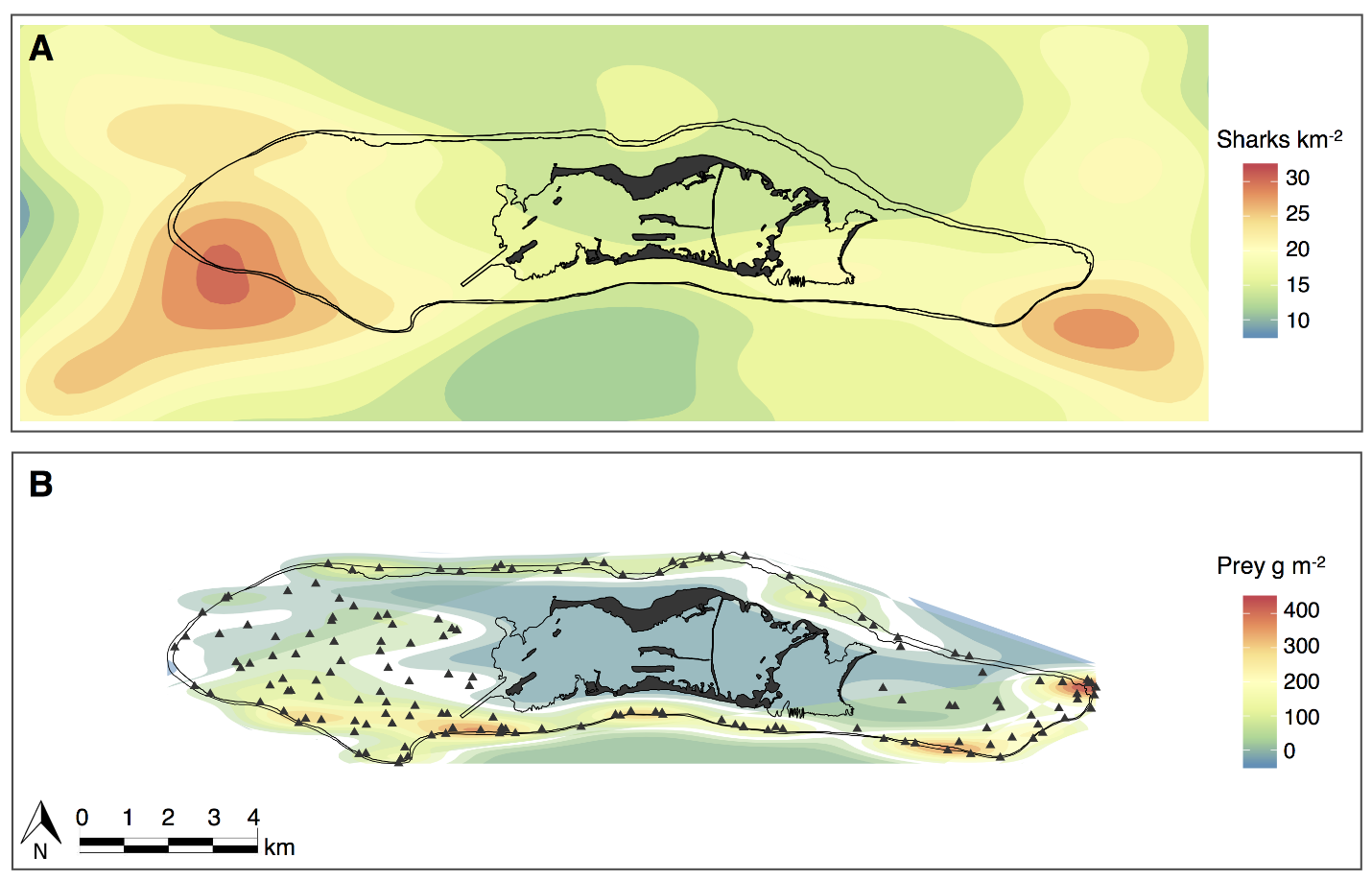


Appendix 8

Methods

Diver spatial point count (SPC) surveys were conducted by NOAA’s Coral Reef Ecosystem Program at Kingman Reef between 2010-2015 (NOAA CREP 2015). For each survey, a pair of divers recorded the number, size, and species of all fishes between 10-60 cm length, observed within a visually estimated cylinder with a 15m diameter over a 5 min interval (NOAA CREP 2015). We only used surveys > 20 m (n=66) to more accurately represent the daytime depth distribution of grey reef sharks.
